# The role of microglia in glaucoma - trigger and potential target

**DOI:** 10.3389/fimmu.2025.1685495

**Published:** 2025-11-11

**Authors:** Liugui Chen, Suyu Yang, Di Wang, Pingping Huang

**Affiliations:** Department of Ophthalmology, Renmin Hospital of Wuhan University, Wuhan, Hubei, China

**Keywords:** glaucoma, high intraocular pressure, microglia, retinal ganglion cell, intercellular communications

## Abstract

Glaucoma is a neurodegenerative disease characterized by the progressive loss of retinal ganglion cell and optic nerve damage. Recent studies have highlighted the pivotal role of microglia in the onset and progression of glaucoma. This review aims to elucidate the key mechanisms of microglial activation in glaucoma and assess its potential as a therapeutic target for novel treatment strategies. Microglia activation in glaucoma is multifactorial, driven by biomechanical, metabolic, and inflammatory signals. Activated microglia contribute to both neuroinflammatory injury and neuroprotective responses. Their interaction with other kinds of cell establishes a dynamic inflammatory signaling network that exacerbates retinal ganglion cell loss. Furthermore, emerging evidence suggests that key targets in microglial activation, such as APOE, LGALS3, CX3CR1, etc. play critical roles in disease progression, revealing promising targets for therapeutic intervention. Microglia act as central regulators of the retinal immune microenvironment in glaucoma. Their dual role in neurotoxicity and neuroprotection is shaped by complex interactions with other kinds of cell. Targeting microglial activation state and restoring metabolic homeostasis represent promising strategies for the development of pressure-independent treatments for glaucoma.

## Introduction

Glaucoma is a group of chronic, progressive neurodegenerative eye diseases characterized by optic nerve damage and visual field defects, and it remains the leading cause of irreversible blindness worldwide (1, 2). A characteristics of glaucoma is the progressive loss of retinal ganglion cell (RGC) and optic nerve degeneration, typically accompanied by elevated intraocular pressure (IOP) (3, 4). It is estimated that approximately 95 million people are affected by glaucoma globally, with at least 10 million experiencing blindness in one eye, and many more suffering from visual impairment that compromises daily functioning (3, 5). Although current treatments primarily focus on lowering IOP, accumulating evidence shows that progressive optic nerve damage still occurs in patients with well-controlled IOP, suggesting that the pathogenesis of glaucoma involves more complex mechanisms beyond pressure elevation alone (6, 7). In recent years, increasing attention has been paid to neuroimmune inflammation in the development of glaucoma, particularly to the activation of microglia, the resident immune cells of the central nervous system, and their close relationship with RGC injury (8, 9).

Microglia are resident innate immune cells in the retina that play essential roles in maintaining tissue homeostasis and responding to injury (10, 11). During the pathogenesis of glaucoma, microglia can be activated by various stimuli such as elevated IOP, axonal injury, and metabolic dysregulation, leading to morphological changes, enhanced secretion of inflammatory mediators, and altered phagocytic activity (12, 13). Activated microglia may induce neuronal apoptosis and axonal degeneration by releasing pro-inflammatory cytokines, reactive oxygen species (ROS), and complement proteins (14–16). In addition, it engage in complex intercellular interactions with other cells, including astrocyte and Müller cell, forming a dynamic network that collectively regulates inflammation, metabolic homeostasis, and the balance between neuroprotection and neurodegeneration.

Recent advances in single-cell transcriptomics, spatial transcriptomics, and metabolomics have enabled researchers to investigate microglia phenotypic and functional heterogeneity in glaucoma with unprecedented resolution (17–19). These technologies have facilitated the identification of microglia subpopulations, signaling pathways, and potential therapeutic targets (20, 21). This review focuses on the mechanisms underlying microglia activation in glaucoma, its interactions with other cells, and its roles in mechanical stress sensing, mitochondrial function, and energy metabolism remodeling. Furthermore, we discuss emerging intervention strategies aimed at modulating microglia function, with the goal of providing theoretical insight for future mechanistic study and therapeutic target discovery in glaucoma.

## Glaucoma

Glaucoma is a common blinding disease characterized by progressive optic nerve damage and visual field loss (4). Based on the anatomical configuration of the anterior chamber angle and the site of aqueous humor outflow obstruction, glaucoma is typically classified into open-angle and angle-closure types (22, 23). Among them, primary open-angle glaucoma is the most prevalent form. Its main pathophysiological mechanism involves structural or functional abnormalities in the aqueous humor outflow pathways, resulting in increased outflow resistance and elevated IOP. In contrast, angle-closure glaucoma is caused by the closure of the anterior chamber angle, which impedes aqueous humor drainage and leads to a rapid rise in IOP. Sustained elevation of IOP can compress the optic nerve head (ONH), damage the axons of RGC, and subsequently trigger progressive RGC degeneration (24).

Although elevated IOP is a major factor causing damage for glaucoma, a subset of patients develops glaucomatous optic neuropathy even when IOP remains within the normal range, a condition referred to as normal tension glaucoma (25, 26). Conversely, some individuals with high IOP do not exhibit detectable optic nerve damage (27, 28). Furthermore, some patients continue to experience disease progression despite adequate IOP control (29, 30). These findings suggest that the pathogenesis of glaucoma involves more than IOP elevation alone and is influenced by a complex interplay of intraocular and systemic factors. Therefore, the development of novel therapeutic strategies that directly protect RGC and preserve visual function through IOP-independent mechanisms is of great clinical importance for improving glaucoma management.

## Microglia in glaucoma

Accumulating evidence suggests that immune and inflammatory responses also play a critical role in its onset and progression (31). In particular, activation of microglia, the resident immune cells of the central nervous system, has emerged as a key pathological feature in glaucoma neurodegeneration.

In the chronic ocular hypertension mouse model, IOP elevation leads to increased microglia numbers and promotes their migration from the inner plexiform layer toward the ganglion cell layer and nerve fiber layer (32, 33). Microglia proliferation begins as early as day 1 after model induction and peaks at week 2 (34, 35). In the unilateral laser-induced ocular hypertension model, microglia activation and migration toward the injury site can be detected within 24 hours of IOP elevation, even in the absence of a significant change in total microglia number (36). Morphologically, activated microglia are characterized by an enlarged soma, retracted processes, and an amoeboid appearance, along with upregulated expression of major histocompatibility complex class II (MHC II), a marker of phagocytic activation (37). In the ONH, such changes are evident as early as day 3 following IOP elevation. By day 7, microglia are widely distributed across the ganglion cell layer and retinal nerve fiber layer, and this activated state persists for at least 2 weeks (35). This finding was further validated in human samples, which showed a significant increase in IBA1 intensity in the retina, although the number of IBA1^+^ microglias did not significantly increase (38). In another human study, numerous amoeboid IBA1^+^ microglias or infiltrating monocytes were observed predominantly along the inner edge of the ILM, where rod-shaped or bipolar IBA1^+^ cells also accumulated (39). This microglial activation was accompanied by a marked upregulation of inflammatory markers and pro-inflammatory cytokines (40). Meanwhile, as a result of RGC degeneration in glaucoma, changes in microglia morphology and gene expression have also been reported in the brain, especially in the dorsolateral geniculate nucleus. These findings suggest that the alteration of these microglia may be a secondary and major adaptive immune response to vision-related neurodegeneration (41). However, the mechanisms underlying glial cell activation in the retina of glaucoma patients and their association with neuronal death remain to be further elucidated. Although single-cell transcriptomic studies have advanced our understanding of the normal human retina, obtaining the retina of glaucoma patients still pose major challenges (17, 42).

As the innate immune cells of the CNS, microglia possess a range of functions, including environmental sensing, phagocytosis of cell debris, and immune regulation (43, 44). Under physiological conditions, microglia exhibit a ramified morphology and continuously monitor their microenvironment via dynamic extension and retraction of their processes. Upon exposure to external stimuli such as elevated IOP or neuronal injury, microglia rapidly transition into an activated state, adopting an amoeboid morphology and exhibiting functional polarization into either pro-inflammatory M1 or anti-inflammatory and M2 phenotypes (45, 46). M1 microglia secrete pro-inflammatory mediators such as tumor necrosis factor-α (TNF-α), interleukin-1β (IL-1β), and inducible nitric oxide synthase (iNOS), thereby amplifying the inflammatory cascade. In contrast, M2 microglia express molecules such as CD206 and IL-10, which contribute to neuroprotection and tissue repair (47). M2 microglia is composed of three distinct subpopulations. It mainly includes the M2a subtype involved in anti-inflammation and tissue repair, the M2b subtype involved in regulating immune responses, and the M2c subtype involved in phagocytosis and immunosuppression (8, 48). In addition, there is a type of microglia that is activated by CSF-1 or IL-34 and is different from the M1 or M2a polarization state, which is defined as M3 type (49). This type of cell may be closely related to the division and prolife. However, there are currently no studies on type M3 glaucoma. Other microglial phenotypes have also been described, including rod-like microglia with elongated somata, limited cytoplasm, and reduced branching, which have been observed in mouse models of glaucoma and are implicated in retinal neurodegeneration (50). Although the M1/M2 classification represents a simplified framework, it remains useful for enhancing our understanding of microglial functional states.

In recent years, advancements in single-cell sequencing have deepened our understanding of the pathological states of microglia in glaucoma. By performing large-scale RNA sequencing on microglia isolated from two distinct models of glaucomatous neurodegeneration, researchers identified a disease-associated microglia (DAM) state, whose transcriptional profile closely resembles that observed in multiple models of neurodegeneration in the brain (51, 52). This state is characterized by the upregulation of secretory molecules such as apolipoprotein E (APOE) and lectin, galactoside binding soluble 3 (LGALS3), pro-inflammatory cytokines including TNF-α and chemokines including C-C motif chemokine ligand 2 (CCL2) (53). In the future, with the application of higher-resolution technologies, our understanding of the spatial and temporal heterogeneity of microglia in glaucoma, as well as their disease-specific features, is expected to deepen (54). Such advances will facilitate the identification of key regulatory factors influencing microglial states and may contribute to the development of targeted therapeutic strategies for glaucoma and other neurodegenerative diseases.

### Neuroprotective microglia in glaucoma

Microglia activation is considered one of the earliest events in glaucomatous neurodegeneration, often preceding detectable RGC loss (55, 56). In experimental glaucoma models, CD206^+^ M2 microglia transiently increase during the early stage, whereas CD86^+^ M1 microglia become predominant at later stage (33, 57). This seems to suggest that microglial activation in the early stage of glaucoma may have a neuroprotective effect, while prolonged or chronic activation can exacerbate neurodegeneration. At this point, the early clearance of microglia in DBA/2J mice with age-related intraocular pressure elevation and glaucomatous neurodegeneration, which shows exacerbated glaucomatous neurodegeneration, also seems to demonstrate the early protective effect against glaucoma (58). Studies have shown that early administration of exogenous IL-4 can prolong the duration of M2 microglial polarization after RIR and effectively improve the loss of RGC in the late stage (57). This suggests that, in addition to inhibiting the pro-inflammatory M1 microglia at later stage, prolonging the presence or activity of M2 microglia may also exert neuroprotective effects.

Following RGC apoptosis, the externalization of phosphatidylserine on the cell membrane acts as a classical “eat-me” signal that activates microglia, thereby inducing their activation and phagocytic response (59). Study has shown that intravitreal injection of apoptotic neurons can trigger microglia activation *in vivo*, suggesting that apoptotic neurons may serve as key stimuli for microglia phenotypic shifts (60). In the early stage of glaucoma, activated M2 microglia clear apoptotic or degenerated RGCs through phagocytosis, thereby maintaining a non-toxic retinal environment and preventing the further spread of inflammation. In addition, M2 microglia can secrete brain-derived neurotrophic factor and other anti-inflammatory cytokines to exert anti-apoptotic effects on RGCs (48).

### Neurotoxic microglia in glaucoma

In later stage of experimental glaucoma, the number of activated M1 microglia gradually increases (33). The activation of microglia in this context primarily exhibits pro-inflammatory and neurotoxic effects. Research has shown that in an experimental mouse model of glaucoma with transient elevation of IOP, pharmacological suppression of microglial activation by minocycline significantly increased RGC survival (61). The protective effect observed with the inhibition of microglial activity, in contrast to the aggravated damage caused by microglial depletion, suggests that the outcomes of modulating microglial activation may vary depending on the timing and the specific intervention strategies applied. At this stage, microglia phagocytose neuronal debris or fragmented DNA, activating intracellular pathways such as NF-κB and cGAS–STING and promoting the release of pro-inflammatory cytokines and exosomes (62). These microglia-derived cytokines and exosomes further amplify inflammation by enhancing microglial migration, phagocytosis, and proliferation, as well as inducing neuronal ROS production and cell death, thereby exacerbating retinal neurodegeneration under elevated IOP conditions (63). It is worth noting that such damage is not limited to RGCs. In animal models of acute or chronic glaucoma, electroretinogram assessments have demonstrated functional impairments in multiple types of retinal cells, including amacrine and bipolar cells (64, 65). Immunohistochemical analyses further corroborate these finding (66). Moreover, although photoreceptor loss is typically not associated with primary open-angle glaucoma, it has been reported in cases of secondary angle-closure glaucoma and in experimental animal models (67–70). Highly activated microglia may therefore contribute to the degeneration and loss of retinal cells beyond RGCs.

Beyond biochemical stimuli, physical factors such as mechanical stretch may also contribute to microglia activation (71). The mechanosensitive ion channel PIEZO1 is highly expressed in microglia cell lines and brain endothelial cells and has been shown to regulate microglia motility and immune responsiveness (72). In monocyte, PIEZO1 mediates calcium influx in response to mechanical stimulation, thereby activating hypoxia inducible factor-1α (HIF-1α)-dependent inflammatory pathways (73). In both *in vitro* and *in vivo* settings, Piezo1 knockout in microglia suppresses LPS-induced expression of inflammatory cytokines, while treatment with the PIEZO1 agonist YODA1 enhances their production (74). Another mechanosensitive and osmotically sensitive ion channel, transient receptor potential vanilloid 4, may also sense extracellular matrix stiffness and regulate microglia inflammatory responses (75, 76). These findings indicate that microglia can sense mechanical stress resulting from elevated IOP and respond with inflammatory activation. ONH is the primary site of early glaucomatous damage. Because RGC axons converge at this region, microglia in the ONH exhibit heightened sensitivity to mechanical deformation and extracellular matrix remodeling compared with Müller cells. Moreover, due to the close crosstalk between microglia and astrocytes, mechanotransduction within ONH astrocytes can trigger their activation and the release of inflammatory mediators, which in turn modulate microglial behavior through intercellular signaling (77). It is important to note that for normal pressure glaucoma, mechanical stress may not play a major role in the triggering of inflammatory damage.RGC loss, progressive axonal degeneration and reactive gliosis were observed in OPTN-E50K knock-in mice, a commonly used animal model of normal stress glaucoma. One possible mechanism is that retinal microglia regulate high levels of apolipoprotein A1 to lead to apoptosis of vascular endothelial cells and reduction in retinal peripapillary vascular density, thereby further augments RGCs damage (78).

**Figure 1:**
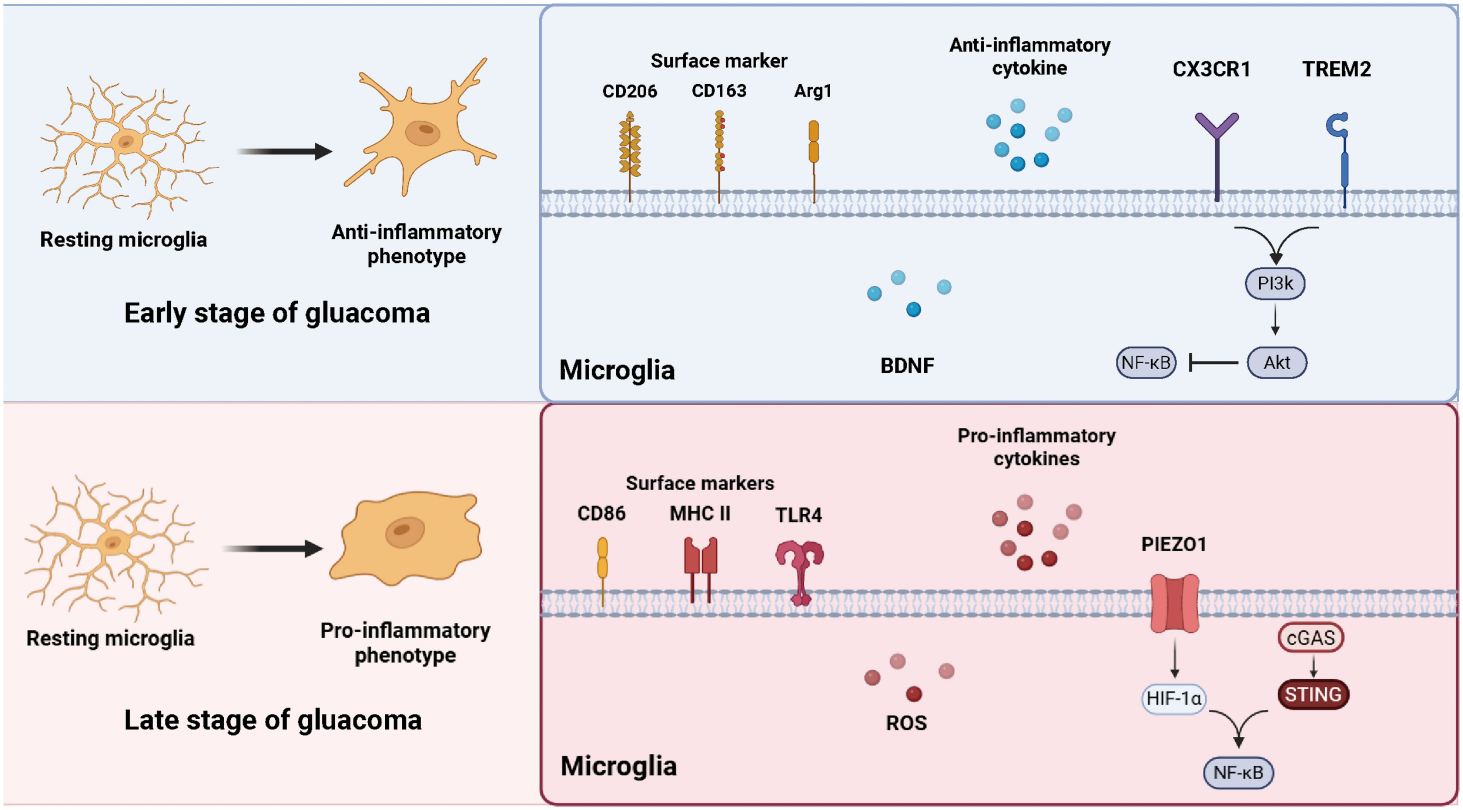
Microglial phenotypic transformation during glaucoma. In the early stage of glaucoma, resting microglia shift toward an anti-inflammatory phenotype, characterized by the expression of surface markers CD206, CD163, and ARG1, and the release of neuroprotective factors such as BDNF, CX3CR1 and TREM2 signaling maintain microglial homeostasis through the PI3K/AKT and NF-κB pathways. In the late stage, microglia adopt a pro-inflammatory phenotype expressing CD86, MHC II, and TLR4, accompanied by increased production of ROS and pro-inflammatory cytokines. External stimulus further triggers the cGAS–STING and HIF-1α–NF-κB pathways, amplifying neuroinflammation and contributing to RGC degeneration. (The figure is created by https://www.biorender.com/).

The integrity of mitochondrial function plays an important role in regulating microglia polarization under inflammatory conditions. Mitochondrial dysfunction is recognized as a key driver of pro-inflammatory microglia phenotypes in various neurodegenerative diseases (79, 80). In DBA/2J mice, microglia exhibit transcriptional signs of metabolic dysregulation prior to axonal degeneration at the ONH, including upregulation of mitochondrial genes involved in oxidative phosphorylation and abnormal expression of genes related to glycolysis, gluconeogenesis, and lipid metabolism (81). Similarly, RNA sequencing of microglia in glaucoma animal models reveals increased expression of Slc16a1, a bidirectional transporter of lactate, pyruvate, and ketone bodies, suggesting heightened metabolic demands and functional plasticity of microglia in early stage of glaucoma (81). This metabolic shift may be associated with the upregulation of HIF-1α, the key glycolytic transcription factor under oxidative stress and inflammatory conditions, as further supported by studies using microbead-induced ocular hypertension mouse models (82). Moreover, mitochondrial fragmentation is enhanced in activated microglia and released extracellularly (83). These extracellular mitochondria may trigger innate immune responses in neighboring astrocyte or serve as signaling molecules in glia-neuron interactions (84). Mitochondrial dysfunction may also contribute to retinal hypoxia and reduced glucose availability, resulting in excessive ROS generation, oxidative stress, and exacerbated RGC damage (85, 86). (**Figure 1**)

## Microglia crosstalk with retinal cells in glaucoma

### Astrocyte

Astrocyte play a vital supportive role for RGC within the retina. They are primarily localized in the nerve fiber layer and ganglion cell layer, where they envelop blood vessels extensively in the ONH region (11). Similar to their functions in the brain, retinal astrocyte contribute to the maintenance of the blood-retinal barrier (BRB) and immune privilege through close interactions with the vasculature (12, 87). They regulate vascular tone and facilitate the transport of metabolic substrates from the bloodstream, thereby supporting the energy demands of RGC axons.

In glaucoma, pro-inflammatory activation of microglia may disrupt astrocyte function through the release of cytokines and other mediators. M1-polarized microglia can secrete inflammatory factors such as complement component C1q, IL-1β, and TNF-α, which collectively drive the transformation of astrocyte into a neurotoxic A1 phenotype, further amplifying neuroinflammation (88). Studies in mouse models of glaucoma have shown that the glucagon-like peptide-1 receptor agonist NLY01 can inhibit the production of these cytokines by microglia and prevent the induction of A1 astrocyte, ultimately protect the RGC. Under homeostatic conditions, both astrocyte and microglia express matrix metalloproteinase-2 primarily at their perivascular endfeet (89). However, upon activation, the expression of matrix metalloproteinase-2 is markedly upregulated, contributing to increased BRB permeability, pathological neovascularization, and glial scar formation, all of which exacerbate retinal injury (90, 91).

### Müller cell

Müller cells are the principal radial glial cells in the retina and play a pivotal role in maintaining retinal homeostasis. Müller cells provide necessary metabolic support and ionic homeostasis for RGCs. In the early stage of glaucoma, muller cells can sense retinal hypoxia earlier, but the activation at this time may still be mainly protective, providing necessary metabolic support for the hypoxic nerve cells (82). In addition, upon activation, Müller cells can release ATP through connexin 43 hemichannels (92, 93). Studies have shown that ATP released by activated Müller cells can trigger microglia activation by stimulating P2X7R on the microglia surface (33). On the day4 after chronic ocular hypertension (COH), activated microglia in branched and amoeba-like shapes were observed. In addition, microglia transfer from the inner/outer plexiform layer of the retina to the GCL of the COH retina (94, 95). Intravitreal injection of the P2X7R agonist BzATP or *in vitro* stimulation with DHPG can increase the migration and proliferation of microglia. In particular, the P2X7R, which is closely associated with inflammatory responses, can be activated by ATP to initiate NOD-like receptor thermal protein domain associated protein 3 (NLRP3) inflammasome assembly and induce the release of multiple pro-inflammatory mediators, such as IL-1β (96–98). It has been demonstrated that pharmacological blocking or knockout of P2X7R delays activation of microglia and RGC death following COH in mice (35). Further evidence supports the involvement of microglial activity in RGC degeneration, as P2X7R knockout has been shown to delay RGC death following optic nerve crush in mice (99).

In normal mice, intravitreal injection of the group I metabotropic glutamate receptor agonist DHPG has been shown to induce Müller cell activation. Following DHPG administration, the expression of translocator protein (TSPO), the markers of microglia activation, gradually increases and becomes significantly elevated after one week. In contrast, inhibition of Müller cell activation using the metabotropic glutamate receptor 5 antagonist MPEP leads to a reduction in TSPO level, along with decreased GFAP expression, suggesting that Müller cell activation may serve as an important upstream event in microglia activation (33).

In addition to inflammatory crosstalk, Müller cells and microglia engage in neuroprotective interactions mediated by neurotrophic factors. Microglia can secrete nerve growth factor and brain-derived neurotrophic factor, which not only exert direct protective effects on RGC but also promote the expression of basic fibroblast growth factor and glial cell line-derived neurotrophic factor in Müller cells, thereby exerting synergistic neuroprotective effects (100–102).

### Monocyte

Both active extravasation and passive leakage of monocyte have been observed in experimental models of glaucoma, closely associated with the breakdown of the BRB (103, 104). Notably, monocyte infiltration has been detected in the ONH region of glaucoma eyes, suggesting that monocyte recruitment may be an early pathological feature of the disease, as documented in several animal models (39, 105–107).

The CCL2 signaling axis plays a critical role in regulating monocyte migration. CCL2 binding to its receptor CCR2 directs monocyte to sites of injury, promoting their infiltration. Elevated levels of CCL2 have been shown to reduce neuronal survival in animal models of glaucoma (108). Conversely, genetic deletion of Ccl2 significantly preserves RGC and reduces the density of retinal myeloid cells, without altering the expression levels of pro-inflammatory cytokines. These findings suggest that monocyte recruitment itself may exert pathogenic effects independent of subsequent inflammatory signaling (109).

## Therapeutic strategies targeting microglia in glaucoma

Currently, regardless of the subtype of glaucoma, clinical treatment primarily focuses on lowering IOP by either reducing aqueous humor production or enhancing its outflow. However, extensive clinical and experimental evidence indicates that IOP-lowering therapy alone is insufficient to halt the progressive visual deterioration of the majority of patients (110). Consequently, there is an urgent need to develop IOP-independent therapeutic strategies for glaucoma.

While mice express a single APOE isoform, humans possess three major alleles (APOE2, APOE3, and APOE4). APOE4 is a well-established genetic risk factor for Alzheimer’s disease, but paradoxically, it has been associated with reduced glaucoma risk in several human studies (111–113). Even under elevated IOP conditions, ApoE4-expressing microglia exhibit robust suppression of pro-inflammatory genes such as Lgals3, Tnf-α, and Ccl2, while maintaining the expression of homeostatic genes like C-X3-C motif chemokine receptor 1 (Cx3cr1) and colony-stimulating factor 1 receptor (Csf1r) (114, 115). Moreover, ApoE is markedly upregulated in phagocytic retinal microglia, and this finding has been validated at the protein level. In wild-type microglia, phagocytic activation induces the upregulation of DAM genes such as Lgals3, Gpnmb, Spp1. In contrast, this phenotype is significantly attenuated in APOE-deficient retinal microglia (60).

LGALS3 is considered a key regulator of microglia activation. It has been shown to directly activate microglia, act as a chemoattractant for monocyte, and serve as a marker of phagocytic states (116, 117). In both the microbead-induced model and DBA/2J mice, Lgals3 expression is upregulated at both the mRNA and protein levels, and is modulated by APOE signaling. In the microbead model of glaucoma, Lgals3 is one of the genes most strongly affected by APOE deficiency (60). Genetic deletion of Lgals3 or pharmacological inhibition using agents such as TD139 significantly protects RGC, even under elevated IOP (60, 118). Although the precise mechanisms underlying the neurotoxicity of LGALS3 remain unclear, it is known to function as an endogenous ligand for toll-like receptor 4, potentially acting upstream of inflammasome activation (119, 120). Furthermore, LGALS3 can bind the receptor, and may serve as a critical molecular bridge for its activation in microglia (121, 122). Collectively, these findings support the involvement of LGALS3 in mediating pathological inflammation and phagocytic responses of microglia in glaucoma neurodegeneration.

Under physiological conditions, the homeostasis of microglia is tightly regulated by a repertoire of signaling molecules that suppress aberrant activation through interactions with microglia surface receptors. Among these, the CX3CL1-CX3CR1 axis is a critical inhibitory pathway. CX3CR1, the receptor for CX3CL1, is predominantly expressed in microglia within ocular tissues (123). Studies have shown that CX3CR1 can suppress the expression of pro-inflammatory cytokines such as IL-1β and CCL2 under homeostatic conditions (124, 125). In animal models of glaucoma, CX3CR1 deficiency lowers the activation threshold of microglia, enhances their pro-inflammatory phenotype, and exacerbates RGC loss in response to transient IOP elevation (61, 126, 127). In the rd10 mouse model of retinal degeneration, CX3CR1 knockout results in increased microglia phagocytic activity and elevated secretion of pro-inflammatory mediators, accelerating photoreceptor degeneration. Conversely, intravitreal administration of recombinant CX3CL1 effectively suppresses abnormal microglia activation, suggesting a potential neuroprotective role of this signaling axis (128, 129). Moreover, the loss of CX3CL1- CX3CR1 signaling can also exacerbates disease pathology in other ocular disease models. For example, in laser-induced choroidal neovascularization model, CX3CR1-deficient mice exhibit more severe phenotypes, including thinning of the outer retina and drusen-like subretinal deposits (125). Similarly, in experimental autoimmune uveitis, CX3CR1 deficiency correlates with increased disease severity (130).

The precise physiological roles of microglia in the retina remain under investigation. Genetic tools targeting CX3CR1 have been used to selectively deplete microglia, revealing that retinal neurons can maintain gross structural integrity and viability in the absence of microglia (131). However, functional assessments showed a gradual reduction in electroretinography amplitudes in response to light stimuli, despite retained visual function. Transmission electron microscopy further revealed dystrophic and morphologically abnormal presynaptic terminals, suggesting that microglia may play a crucial role in maintaining mature retinal synaptic integrity.

Additional studies using the CSF1R inhibitor PLX5622 to deplete microglia showed that PLX5622 treatment alone does not impair RGC function. However, in ischemia reperfusion injury models, microglia depletion significantly attenuated IR-induced neuroinflammation and inner BRB disruption (132). In diabetic retinopathy models, approximately two months of PLX5622 treatment also mitigated neurodegenerative and vascular changes (133). In contrast, in models of acute optic nerve crush injury, microglia depletion via PLX5622 had no significant impact on RGC degeneration (134). It suggests that microglia may play a more prominent role in responding to extrinsic stressors (e.g., ischemia, elevated IOP, or metabolic dysregulation) than in direct RGC injury.

At the molecular level, genome-wide association studies have identified common variants near the ABCA1 gene as being associated with increased glaucoma susceptibility (135, 136). ABCA1, a cholesterol efflux pump, plays a vital role in maintaining lipid homeostasis and modulating inflammatory responses. Deficiency of ABCA1 has been linked to retinal neurodegeneration (137). In a mouse model of acute IOP elevation-induced ischemia reperfusion injury, elevated IOP promotes ubiquitin-mediated degradation of ABCA1. This in turn impairs membrane translocation of annexin A1, facilitates microglia activation, and contributes to RGC apoptosis (138).

## Conclusion

Glaucoma is a multifactorial neurodegenerative disorder, the progression of which involves a complex interplay of cellular and molecular mechanisms. Among these, microglia activation play a central role in shaping the retinal inflammatory microenvironment and mediating RGC damage. Accumulating evidence indicates that microglia contribute not only through the release of pro-inflammatory and neurotoxic mediators, but also via intricate crosstalk with other kinds of cells, collectively forming a dynamic network of metabolism and homeostasis regulation.

This network is modulated by multiple pathological cues, including cell death, mechanical stress and mitochondrial dysfunction. These findings underscore the importance of shifting from a reductionist that approach focused on individual cell types or signaling pathways to a systems-level perspective that emphasizes the coordinated interactions among different cell populations.

Advances in single cell transcriptomics, spatial omics, and metabolomics offer powerful tools to unravel the spatiotemporal dynamics of cell networks in glaucoma. Integrating these technologies holds great promise for deciphering the molecular logic underlying glial reprogramming and for identifying novel therapeutic strategies aimed at microglia functional modulation, inflammation resolution, and metabolic intervention in glaucoma.
